# *Saccharomyces cerevisiae* Laryngitis and Oral Lesions in a Patient with Laryngeal Carcinoma

**DOI:** 10.1155/2017/2941527

**Published:** 2017-11-26

**Authors:** Jumanah N. Algazaq, Kevan Akrami, Fernando Martinez, Allen McCutchan, Ajay R. Bharti

**Affiliations:** ^1^Department of Health Sciences International, The University of California, San Diego, CA, USA; ^2^Division of Infectious Diseases, The University of California, San Diego, CA, USA; ^3^Division of Pathology, University of Texas MD Anderson Cancer Center, Houston, TX, USA

## Abstract

*Saccharomyces cerevisiae* is increasingly being promoted as a nutritional supplement by health food enthusiasts and is also recommended as prophylaxis against antibiotic-associated diarrhea. However, severe opportunistic infections due to *S. cerevisiae* have been reported in patients with chronic disease, cancer, and immunosuppression. Fungemia, endocarditis, pneumonia, peritonitis, urinary tract infections, skin infections, and esophagitis have been described. It is important to consider infections due to *S. cerevisiae* in appropriate clinical settings. Here, we describe the first case of *S. cerevisiae* laryngitis in a patient with a history of laryngeal carcinoma who also had oral lesions.

## 1. Introduction

*Saccharomyces* species are ubiquitous yeasts that have been used for centuries in baking and the production of beer and wine and are commonly known as baker's or brewer's yeast. *Saccharomyces cerevisiae* is used as a probiotic for prophylaxis of antibiotic-associated diarrhea [1] and is also promoted as a nutritional supplement by health food enthusiasts. It is part of the normal flora of the oral, gastrointestinal, and respiratory tracts and the vaginal mucosa [2]. Severe opportunistic infections due to *S. cerevisiae* have been reported in patients with chronic disease, cancer, and immunosuppression presenting as fungemia, endocarditis, pneumonia, peritonitis, urinary tract infections, skin infections, and esophagitis [3]. Here, we describe the first case of *S. cerevisiae* laryngitis in a patient with a history of laryngeal carcinoma who also had oral lesions.

## 2. Case Report

**Figure 1:**
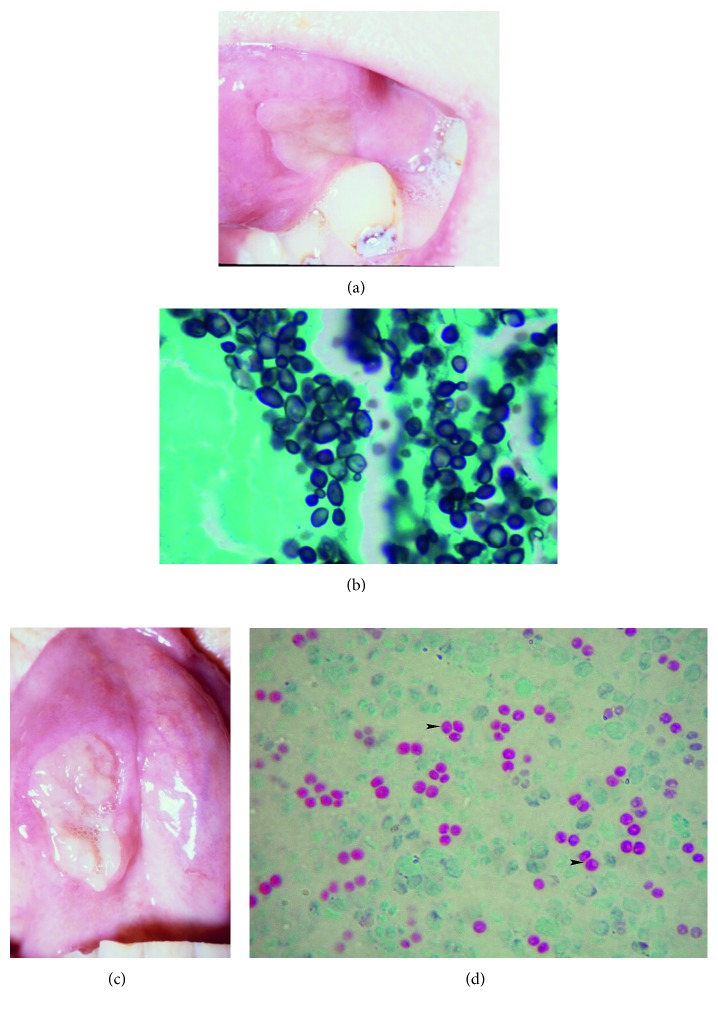
(a, b) Lesions on the tongue (4 × 2 cm and 3 × 2 cm) with slightly rolled and raised edges. (c) High-power view of a Gomori methenamine silver stain (GMS). Biopsy from ulcerated vocal cord lesion demonstrates spherical to oval yeast cells, some of which are budding. (d) High-power view of a Kinyoun stain. Round ascospores (arrowhead) inside the asci that are characteristic of *S. cerevisiae*.

A 63-year-old Caucasian female with squamous cell carcinoma of the vocal cords developed a small grayish lesion on the ventral surface of her tongue one month after local external radiation therapy (XRT). It gradually increased in size and the patient noticed 2 new lesions (3 × 2 cm and 4 × 2 cm), one on either side of the tongue (Figures 1 and 1). The lesions had well-demarcated margins with slightly raised and rolled edges, without bleeding or discharge, and were initially painless, but later became extremely painful. Biopsy did not reveal any malignancy. Symptoms progressed and she developed shortness of breath, stridor, and hoarseness of voice that prompted evaluation by an otolaryngologist. Laryngoscopy revealed grayish exudates on both vocal cords that were similar in appearance to the oral lesions. No oropharyngeal mucositis was seen. Complete blood cell count at the time of presentation did not reveal any abnormalities. Information regarding mucositis or leukopenia immediately following XRT was not available. The patient was started empirically on oral fluconazole 400 mg/day for presumed candidal infection. There was no improvement despite 2 weeks of therapy, and she presented to the emergency room with severe difficulty in breathing. Repeat laryngoscopy revealed worsening of the grayish exudates on the vocal cords, vocal cord edema, and narrowing of the airway that required tracheostomy.

Right vocal cord biopsy showed ulcerated squamous mucosa with polymorphonuclear leukocytes, and monocytes and macrophages indicating acute and chronic inflammation. No stromal fibrosis to indicate effects of radiation therapy was noted. Abundant extracellular oval budding yeast forms were seen on hematoxylin and eosin and Grocott's methenamine silver stains (Figure 1). Tissue culture on Sabouraud dextrose agar at 30°C grew a creamy moist colony that was evident after 72 hours. This was then placed on Cornmeal agar, and a Vitek card identified it as *Saccharomyces cerevisiae*. Microscopic examination revealed multilateral ellipsoidal budding yeasts without a capsule with a largest diameter of 7–9 μm and occasional pseudohyphae. Kinyoun stain revealed oval asci with 1–4 ascospores inside, a characteristic finding of *Saccharomyces* genus (Figure 1). Using the broth microdilution method of the Clinical and Laboratory Standards Institute [4], the isolate was found to have fluconazole MIC ≤ 8 μg/mL.

Given lack of response to oral fluconazole 400 mg/day after 2 weeks despite low fluconazole MIC, the dose was increased to 800 mg/day. Significant improvement in the tongue lesions was seen within 1 week with 50% reduction in the size of the lesions. After 4 weeks, one of the tongue lesions had resolved completely, and the other showed 90% clearance. Repeat laryngoscopy also showed significant improvement, and fluconazole was continued for 6 weeks until the oral lesions had resolved completely.

## 3. Discussion

To our knowledge, this is the first case report of a patient with laryngitis due to *S. cerevisiae* who also had oral lesions presumably due to the same infectious agent given their similar appearance. *Saccharomyces* genus was identified based on microscopy, and *S. cerevisiae* was confirmed by Vitek. Acute and chronic inflammation seen on the vocal cord biopsy indicated that it was a true infection and not just colonization. Inflammation combined with lack of fibrosis makes it unlikely that the pathological changes were due to radiation therapy.

Head and neck XRT is associated with increased risk of oral fungal infections. Oropharyngitis has been reported in a 65-year-old male, but this was after receiving both chemotherapy and XRT for head and neck carcinoma [5]. Interestingly, the prevalence of oral candidiasis during head and neck XRT (37.4%) is similar to that during chemotherapy (38%) [6]. But there are no reports on *Saccharomyces* infection or colonization following head and neck XRT. Possible mechanisms for increased risk of oral fungal infections, including *Saccharomyces*, may be hyposalivation and local tissue damage although these were not found in our patient.

Upon reviewing 22 documented cases of *S. cerevisiae* infections in patients with cancer, we found four where on probiotics while on chemotherapy that have been the source of infection [5, 7]. Other possible sources include locally brewed beer [8] and, as in our patient, grapes [9]. A detailed history of probiotic supplement intake, food, and drink may reveal possible sources in patients with *S. cerevisiae* infections.

*Saccharomyces boulardii*, a component of several probiotic supplements, was initially classified as a novel species of the genus *Saccharomyces*. Although there are some physiological differences, comparative gene analysis has characterized it as a strain of *S. cerevisiae* [10]. Probiotics labeled as containing *S. boulardii* have resulted in fungemia due to yeast identified as *S. cerevisiae* var. *boulardii* [11]. For clinical purposes, the two can be considered to be the same.

Diagnosis of *S. cerevisiae* is facilitated by matrix-assisted laser desorption/ionization time-of-flight mass spectrometry (MALDI-TOF MS) for rapid and accurate identification of *S. cerevisiae* [12]. Cultures should be obtained in high-risk patients with fever of unknown origin, leukocytosis despite antibiotic therapy, and a history of probiotic use or beer consumption. Our case is from 2003 when MALDI-TOF MS was unavailable and diagnosis was based on culture and special stains showing the characteristic ascospores (Figure 1) and Vitek.

*S. cerevisiae* is consistently susceptible to amphotericin B (0.5–1 mg/mL), but mean MIC^90^ and ranges of susceptibility to fluconazole (2/0.1–128) and itraconazole (0.5/0.015–64) are much more variable [3, 13]. However, no therapeutic failures have been clearly attributable to high fluconazole MIC. In our patient, despite low fluconazole MIC, a higher dose of 800 mg/day was required. In cases with severe infection, combination therapy with amphotericin B and fluconazole is associated with better outcomes compared to monotherapy with amphotericin B alone [3]. Poor outcomes were mostly associated with older age, delayed detection and treatment, disseminated infection, and associated critical conditions such as pancreatic cancer, aplastic anemia, myelodysplastic syndrome, relapsing acute lymphocytic leukemia, cardiac surgery, and neurosurgery. Management should include discontinuation of probiotics containing *Saccharomyces*, central venous catheter (CVC) removal, and consideration of combination antifungal therapy.

In conclusion, *S. cerevisiae* should be considered as a potential pathogen when recovered from patients with cancer, immunosuppression, presence of CVC, and other critical illnesses. In addition, since contaminated vascular catheters may be the point of entry in some cases, probiotic treatments containing *Saccharomyces* should be carefully handled to avoid aerosolization and cross contamination [14]. In case of fever of unknown origin in high-risk patients, fungal cultures must be considered and performed early to avoid a delay in treatment. Finally, in cases of documented fungemia, management should include antifungals, removal of CVC, and discontinuation of probiotics. With rise in probiotic use [15], more *S. cerevisiae* infections can be expected and should be considered in the differential diagnosis.
